# The Association Between Mental Health Indices and the Chronotypes Measured by Single-Item Chronotyping in Young Adults

**DOI:** 10.3390/jcm14134447

**Published:** 2025-06-23

**Authors:** Katarzyna Nowakowska-Domagała, Małgorzata Juraś-Darowny, Jacek Koprowicz, Aleksandra Lewandowska, Tadeusz Pietras, Łukasz Mokros

**Affiliations:** 1 Institute of Psychology, Faculty of Educational Sciences, University of Lodz, Rodziny Scheiblerów 2, 90-128 Lodz, Poland; malgorzata.juras.darowny@edu.uni.lodz.pl; 2 Mental Health Clinic, National Institute of Medicine of the Ministry of the Interior and Administration in Warsaw, 13 Wołoska Street, 02-507 Warsaw, Poland; jacek.koprowicz@pimmswia.gov.pl; 3 J. Babiński Specialist Psychiatric Health Care Team, Psychiatric Ward for Children, Aleksandrowska 159, 02-229 Lodz, Poland; aleksandra_lewandowska@poczta.onet.pl; 4 Department of Clinical Pharmacology, Medical University of Lodz, 22 Kopcińskiego Street, 90-153 Lodz, Poland; tadeusz.pietras@umed.lodz.pl; 5 Department of Child and Adolescent Psychiatry, Medical University of Lodz, Czechoslowacka Str 8/10, 92-216 Lodz, Poland; lukasz.mokros@umed.lodz.pl

**Keywords:** chronotype, SIC, mental health, suicide, suicidal behavior, suicide acceptance, sleepy daytime type, the moderately active type

## Abstract

**Background:** Chronotype reflects individual variations in daily activity and sleep patterns, influenced by underlying circadian rhythms. While chronotype is often reduced to the morningness–eveningness spectrum, recent evidence suggests more diverse circadian typologies. Chronotype is linked to mental health, frequently associated with psychiatric disorders such as depression and suicide. This study aims to examine differences among six chronotypes (as defined by Single-Item Chronotyping) in mental health outcomes, including depression, anxiety, interpersonal relations, general functioning, suicidal behavior, and suicide acceptance. **Methods:** The study sample consisted of 306 young adults. Chronotype was determined using the Polish version of Single-Item Chronotyping (SIC). Mental health was assessed with the 30-item General Health Questionnaire (GHQ-30), which evaluates three dimensions: depression and anxiety, interpersonal relations, and general functioning. The Suicide Behavior Questionnaire (SBQ-R) measured past and potential future suicidal tendencies, while the Suicide Acceptance Questionnaire (SAQ) assessed attitudes toward the act of suicide. **Results:** The “daytime sleepy” and “moderately active” chronotypes were identified as at higher risk for mental health issues. These types exhibited greater levels of depression and anxiety, more general dysfunction, and a higher risk of suicidal behavior. The “daytime sleepy” type also experienced more interpersonal relationship difficulties compared to the “daytime active” type. **Conclusions:** Recognizing the “daytime sleepy” and “moderately active” types as risk factors highlights the importance of considering chronotype in mental health assessments. The SIC provides a concise method for monitoring circadian rhythm changes during treatment, facilitating tailored interventions such as modifying treatment schedules or lifestyle adjustments in alignment with an individual’s circadian rhythm.

## 1. Introduction

Chronotype reflects individual differences in diurnal activity and sleep patterns, serving as a manifestation of the underlying circadian rhythm, also known as the *circadian pacemaker* [1,2]; this is considered to be one of two processes regulating the sleep–wake pattern, together with the homeostatic process of sleep debt [3]. Traditionally, chronotype has been assessed through self-reported sleep patterns; however, this approach often overlooks critical physiological and contextual factors such as daytime levels of alertness and energy, hormonal cycles, social habits, and environmental influences [4]. A comprehensive understanding of chronotype requires the integration of its biological foundations with its social and ecological context. Often conceptualized along a continuum from morningness to eveningness, chronotype is closely linked to sleep timing and quality, with evening types more prone to sleep debt and social jetlag due to circadian misalignment [5,6]. Based on the two-process model of sleep–wake regulation, the dysregulation of the biological circadian rhythm with regard to its social counterpart (i.e., disruption of the circadian pacemaker) appears to be separate from the sleep debt itself (i.e., disruption in the homeostatic process). Nevertheless, it may still result in a diminished amount of sleep and thus cause the aforementioned consequences [3,7].

Despite the bespoken frequent reduction of chronotype to the morningness–eveningness spectrum [8], the latest evidence seems to confirm the existence of more diverse circadian typology [9]. Originally developed by Putilov et al. on the basis of a series of research investigating 24 h alertness–sleepiness patterns [10], the LIVEMAN classification consists of morning and evening types, highly active and moderately active (i.e., energetic vs. lethargic), and daytime active and daytime sleepy (i.e., afternoon vs. napper). By devising a novel and effective tool—Single-Item Chronotyping (SIC), Putilov [11] proposed a simple, yet accurate in reflecting the actual diurnal activity patterns, approach to self-assessment of chronotype.

The study of chronopsychology and biological rhythms has been based on a number of acclaimed questionnaires, such as the Morningness–Eveningness Questionnaire (MEQ; [12]) and the Composite Scale of Morningness (CSM; [13]). While these tools are generally highly regarded and have undeniably contributed to the advancement of the discipline, they also present significant limitations. Not only do they encompass mainly the morning or evening aspect of chronotype, but also completing them is quite time-consuming when considered in the context of specialist consultation. As such, they could not act as operative screening tools, allowing us to assess and monitor chronotype and its potential alterations.

Circadian rhythm and its expression, in the form of chronotype, are closely linked to mental health and are often associated with psychiatric disorders and symptoms such as depression and suicidal behavior [14,15]. However, a significant body of research focuses on the morning–evening preference, indicating eveningness as a global indicator of mental health risk [14,16,17]. This association is not entirely direct; rather, it is largely mediated by sleep quality—individuals with an evening chronotype often report shorter sleep duration, irregular sleep–wake schedules, and increased daytime dysfunction, all of which contribute to poorer mental health outcomes [18]. While the morningness–eveningness continuum provides valuable insights, it captures only a narrow dimension of circadian variability. Chronotype is a multifaceted construct encompassing not just temporal preference but also physiological, behavioral, and contextual factors. Relying solely on morning–evening typology may overlook differences between individuals and situational influences that shape circadian functioning. This appears particularly relevant in the context of recent research indicating evening types are prone to negative self-image and social perception [19,20].

Therefore, expanding chronotype assessment beyond traditional typologies may offer a more nuanced and clinically relevant understanding. In this context, Single-Item Chronotyping emerges as a promising, time-efficient tool for capturing broader aspects of circadian orientation and monitoring its potential alterations in everyday or clinical settings. Such an approach could enhance the precision of chronotype-related mental health screening and interventions.

Consequently, it is necessary to analyze mental health indices and identify potential risks associated with LIVEMAN chronotypes. The aim of this study is to explore differences between the six chronotypes (defined by SIC) in general mental health (depression and anxiety symptoms, interpersonal relations, and general functioning), suicidal behavior, and acceptance of suicide. The study also includes substance use, which is strongly linked to circadian rhythm disturbances [21].

## 2. Materials and Methods

### 2.1. Subjects

The initial sample consisted of 313 participants—students aged 18 to 48 years (M = 21.99; SD = 3.21). To ensure the study focused specifically on young adults without psychiatric diagnoses, individuals over the age of 30 and those with a diagnosed mental illness were excluded. Consequently, the final sample comprised 306 young adults aged 18–30 years, including 204 (65.8%) women, 101 (32.6%) men, and a participant who chose not to identify with either option (0.3%).

### 2.2. Procedures

The study was carried out according to institutional and national ethical standards and in accordance with the Declaration of Helsinki guidelines. Due to the observational nature of the study and the involvement of noninvasive measures (namely, only self-report questionnaires), ethical review and approval were waived for this study by the Bioethics Committee at the Medical University of Lodz.

Participants were recruited via Facebook, specifically through groups targeting young adults and university students. A snowball sampling method was employed, whereby initial participants were encouraged to share the study information within their social networks to facilitate broader dissemination, which contributed to a gradual increase in sample size.

Individuals who expressed interest were directed to an online research platform, where they were first presented with an information sheet outlining the study details. Informed consent was obtained electronically. Participants were required to read the study description and confirm their willingness to participate by selecting the appropriate checkbox in the online form. Only after providing consent were they granted access to the set of research instruments.

While completing the questionnaire, the participants confirmed in the form that they provided voluntary consent to participate in the study, were informed about its purpose, and acknowledged that they had the right to withdraw from the study at any point without incurring any consequences.

The questionnaires were administered in electronic format, allowing participants to complete them at a time and place of their choosing. The study was conducted anonymously, with an average completion time of approximately 20 min. Participation was voluntary, and respondents were informed of their right to withdraw from the study at any point without providing a reason. Upon completion, participants received a debriefing message, including a note of appreciation for their participation and the researchers’ contact information for any inquiries related to the study.

Participants completed a set of the following questionnaires:

- Single-Item Chronotyping (SIC)

The chronotype was evaluated using the Polish version of Single-Item Chronotyping [9], originally devised by Putilov, Sveshnikov, Puchkova et al. (2021) [11]. This tool consists of six graphs referring to patterns of changes in alertness during the day (in the morning, midday, and evening), according to the LIVEMAN classification [10]. The external validity of SIC was confirmed by the authors [9].

- General Health Questionnaire 30-item version (GHQ-30)

The 30-item version of the General Health Questionnaire (GHQ-30) served as an indicator of mental health in the sample. Originally developed by Goldberg as a screening tool [22], GHQ has since been elaborated, adapted into different languages, and widely used in both research and practice. The Polish adaptation of GHQ-30 [23,24] was incorporated into this study. It reflects general mental health through three factors: depression and anxiety, interpersonal relations, and general functioning [25], with Cronbach’s alpha values of 0.901, 0.713, and 0.843, respectively. The current study uses the original terminology when referring to the names of the GHQ-30 scales. However, the results should be, and will be, interpreted as indicating the presence of symptoms of depression and anxiety.

- Suicidal Behavior Questionnaire-Revised (SBQ-R) and The Suicide Acceptance Questionnaire (SAQ)

Both suicidal behavior and acceptance of suicide were assessed. First, the Suicide Behavior Questionnaire (SBQ-R) [26] in the Polish adaptation of Chodkiewicz and Gruszczynska (2020) [27] is considered. It consists of four questions related to both the retrospective and prospective aspects of suicidal tendencies. In the current study, Cronbach’s alpha value was 0.819, reflecting satisfactory internal consistency and overall good psychometric qualities [27]. Secondly, the Suicide Acceptance Questionnaire (SAQ) developed by Stecz et al. (2020) [28] was used to measure the acceptance of the suicide act itself. It consists of 10 items that demonstrate good psychometric attributes, with a Cronbach’s alpha value of 0.810 in this study.

Additionally, participants answered questions concerning substance use (regarding the number of units of alcohol consumed, measured on a Likert scale from 1—below one unit per week, to 5—above 20 units per week).

### 2.3. Statistical Analysis

Statistical analysis was performed in JASP 0.17.2.1. The prevalence of continuous variables, mean, minimum, and maximum values, and standard deviation were presented, and the distribution was assessed using the Shapiro–Wilk W test and analysis of the Q-Q plots of the residuals. The prevalence of nominal variables was presented as the number of observations and the percentage.

For continuous variables, differences between chronotypes defined by the SIC were assessed using one-way ANOVA. The homogeneity of variance between groups was tested using the Levene test. For categorical variables, the Chi-square test and the Kruskal–Wallis test were used. To analyze significant differences between groups, post hoc comparisons were performed using Tukey’s test, with *p*-values adjusted for multiple comparisons using the Bonferroni correction. To assess the effect size, eta squared (η^2^) was used for the analysis of variance (ANOVA) and Kruskal–Wallis test, and Cramér’s V was applied for the chi-square tests. The level of significance was established at alpha = 0.05.

## 3. Results

Statistically significant differences were observed across chronotype groups for all GHQ-30 subscales, SBQ-R, and SAQ scores, with effect sizes ranging from small to moderate (η^2^ = 0.055–0.079). No significant differences were observed in relation to alcohol consumption or other psychoactive substance use. A detailed summary of the analysis, including post hoc results, is presented in Table 1.

Based on the results, the daytime sleepy type was found to be characterized by higher scores for the GHQ-30 depression and anxiety and general functioning scales compared to the highly active type; it also demonstrated higher scores in the GHQ-30 interpersonal relations scale and SBQ-R compared to the daytime active type, and a higher SBQ-R score compared to the morning type. Similarly, the moderately active type demonstrated higher scores in all GHQ-30 scales compared to the highly active type, higher scores in the GHQ-30 interpersonal relations and general functioning scales, as well as the SBQ-R, compared to the daytime active type, and higher SBQ-R scores compared to the morning type.

Importantly, substantial differences emerged in psychoactive substance use, with the highest usage reported among participants classified as daytime sleepy (76%) and daytime active (72%), and the lowest among morning types (10%). These differences were highly significant (*p* < 0.001) with a large effect size (V = 0.54). Additionally, significant differences were found in sex distribution across chronotypes, with a moderate effect size (V = 0.23). Age differences across chronotypes were not statistically significant, with a very small effect size (η^2^ = 0.020). When comparing the proportions of the types between the sex groups, there were more highly active ones (21 vs. 8% within the sex), more daytime active (28 vs. 21%), less evening types (17 vs. 23%), and less daytime sleepy types (11 vs. 22%) among men than women. In addition, the men and women demonstrated a similar prevalence of morning (8 vs. 11%) and moderately active types (15 vs. 15%).

## 4. Discussion

The purpose of this study was to evaluate the differences between the groups regarding mental health in six chronotypes defined by SIC. The analysis included the severity of depression and anxiety symptoms, interpersonal relations and general functioning, suicidal behavior, and acceptance of suicide. Significant differences between the chronotypes were revealed in all the factors mentioned above, except for acceptance of suicide.

Based on the results, despite the usual portrayal of the evening chronotype as a risk factor, among SIC chronotypes, the evening type was not found to be associated with poorer mental health indices. However, two types emerged as potentially burdened with some risks: daytime sleepy and moderately active types.

The daytime sleepy type showed more prevalent symptoms of depression and anxiety and general disturbances in functioning compared to the highly active type, as well as more difficulties with interpersonal relationships compared to the daytime active type. In comparison to the morning type and the daytime active type, the daytime sleepy type was also characterized by a higher risk of suicidal behavior.

The moderately active type exhibited more depression and anxiety symptoms compared to the highly active type, and more general disturbances of functioning and difficulties in interpersonal relationships compared to the highly active type and the daytime active type. It was also characterized by a higher risk of suicidal behavior in comparison to the morning type and the daytime active type.

The current results are partially in line with those of Putilov et al. on the significance of SIC, who found that daytime sleepy, moderately active, and evening types had less healthy sleep, mood, behavior, and habits than daytime active, vigilant, and morning types [10].

Due to the scarcity of research into the LIVEMAN typology, direct comparisons with previous data are limited. However, when considering the key features of the daytime sleepy and moderately active types, i.e., daytime sleepiness, amplitude of the circadian rhythm, and daytime napping, some similarities can be found in existing literature. Liu et al. (2022) [29] reported that among adolescents, excessive daytime sleepiness was associated with increased risk of suicidal behaviors, independent of sleep duration and social jetlag. Similarly, Shepard et al. (2023) [30] concluded that daytime sleepiness predicted suicide risk above and beyond symptoms of anxiety, depression, and major sleep medications among adult psychiatric inpatients. In a recent meta-analysis, Walsh et al. (2024) [31] found a low amplitude of circadian rhythm (particularly characteristic of the daytime sleepy type) to be related to suicidal thoughts and behaviors, with a strong effect size. Excessive daytime sleepiness has been repeatedly linked to depressive and anxiety symptoms, as recently confirmed by Perotta et al. (2021) [32] among medical students. Daytime napping was confirmed to be a risk factor for depression in a meta-analysis by Li et al. (2022) [33]. Also, excessive daytime sleepiness predicted diminished engagement in social activities [34].

However, our present findings do not suggest that the evening type is significantly associated with poor mental health indices in the current study. This may appear inconsistent with previous research, which indicates mostly that eveningness is a risk factor for depressive disorders, substance use disorders, or anxiety symptoms [6]. The little existing research into the LIVEMAN typology has also indicated that eveningness has negative sequelae [10]. This finding may be specific to the studied population, namely, young adults, for whom the sleep quality has been found to be closely associated with mental and physical health than chronotype or sleep duration: Daytime sleepiness or napping may in turn be indicative of poor sleep quality [35]. Also, it is possible that the effect associated with the evening type is not absent, but merely less noticeable than those of the daytime sleepy and moderately active types; as such, the current results would require verification in a larger sample. Additionally, this may also highlight the importance of the energy or alertness level during the daytime, rather than during morning or evening, when most social activity happens; this is especially the case when considering that up to 60% of young adults belong to an *intermediate* group, i.e., being neither definitely morning nor definitely evening types, in the unidimensional circadian preference model [36]. In this context, it should be noted that the graph of the SIC indicates that the evening type has an intermediate, not low energy/alertness in the daytime. This may affect the choice of the circadian preference by the participants, since differences in the SIC distribution have been found between the text and graph versions of the SIC [11].

This highlights the importance of using comprehensive and multidimensional approaches when evaluating chronotypes to capture the nuances of individual circadian patterns. After all, levels of alertness during the day, not only in the morning and evening, can vary [37,38,39].

The results obtained in this study suggest that of the six chronotypes of the LIVEMAN typology, the sleepy daytime type and the moderately active type emerge as risk types in relation to general mental health and suicidal behavior. Regardless of the nature of these relationships, the two types can thus indicate the presence of certain symptoms and/or inclinations towards certain behavior. A shift toward these types may, in turn, imply a concurrent shift toward symptoms. Hasan et al. (2022) [40] report that a change in chronotype toward earlier sleep–wake timing was associated with beneficial effects such as positive mood and increased well-being. Conversely, the negative changes may occur in specific chronotypes, further underscoring the relevance of understanding individual circadian patterns.

Since chronotype assessment using SIC is based only on one question, it makes the method concise and efficient as a screening tool, allowing one to monitor changes in chronotype and circadian rhythm during treatment. Although the MEQ requires approximately 10–15 min to complete and the CSM 5 min, the SIC can be administered in less than 1 min. Furthermore, it could also prove useful when measuring the effects of interventions targeting the chronotype.

Interventions aiming to shift functioning toward earlier patterns, more aligned with social demands, were shown to have a positive impact on mental health, even in a healthy population [41]. Such interventions target not only sleep parameters (e.g., wake-up time, sleep onset) but also caffeine intake, diet, exercise, and naps, resulting in “resetting” biological clocks with the use of behavioral methods [41]. However, what seems to lie at the core of beneficial effects is the alignment of circadian rhythm with sleep–work schedule, as a similar research in which participants slept during the day observed positive impact of interventions using light exposure and melatonin [42]. Recognizing the importance of circadian rhythm can promote circadian medicine. This approach to healthcare proposes not only evaluating and monitoring circadian rhythm dysregulations but also adjusting the treatment administration schedule or lifestyle modifications to individual circadian characteristics [43].

The identification of the sleepy daytime type and the moderately active type as potentially burdened with certain risks highlights the importance of considering different chronotypes when assessing mental health outcomes.

Since mental health outcomes may differ for men and women [44], the sex differences in the prevalence of the LIVEMAN also deserve a comment. The proportions were similar to those observed for the graph version of SIC in the study by Putilov, Sveshnikov, Puchkova, et al. (2021) [11]. A particular difference was seen in the case of the highly active type (dominant among men) and the daytime sleepy type (dominant among women). In studies employing the unidimensional concept of chronotype, young women tend to be more morning-oriented than men [45]; however, no such trend was observed in the present study. This may be due to the addition of the other four types. Despite the lack of comparable research currently available, recent works indicate that excessive daytime sleepiness may be more prevalent among female than male students [46]. However, it appears that the menstrual cycle in women may play a role in their biological and social circadian rhythms, and as such, future studies should consider circamensual rhythmicity as a contributing factor [47].

While our findings have potential clinical significance, it is important to acknowledge the limitations of this study. Although commonly used, self-reported techniques can reflect the convictions and beliefs of participants, rather than actual behavior [14]. To mitigate the impact of this limitation, the methods included are validated and recognized psychometric tools. Furthermore, the study did not account for the influence of specific work schedules, and the sample consisted of young adults, a group that may not yet have experienced some cumulative effects of chronotype [48]. Also, it should be considered that sleep quality was not included as a variable in this analysis. It should be noted that sleep homeostatic regulation is a process separate from the circadian pacemaker [3] and that the aim of the study was to assess the link of the LIVEMAN typology to the mental health (although the depression and anxiety symptoms scale of GHQ-30 comprises questions about sleep disturbances) [25]. Also, while co-administration of MEQ or similar scales can provide additional information and thus translation between scales, the study did not perform any assessment of the association between SIC and other measures of circadian preference (e.g., MEQ). However, while this was not the purpose of this study, similar analyses have been performed in previous research [9]. Lack of a direct comparison between the LIVEMAN typology and MEQ does not allow to decide whether the disagreement between the LIVEMAN “evening type” and MEQ eveningness reflects differences in circadian phenotypes or the differing sensitivities of those scales. For example, it may be speculated that the “daytime sleepy type” is a facet of MEQ eveningness. Future studies should use both SIC and MEQ (or a similar questionnaire) to deepen the understanding of the phenomenon. Lastly, while SIC introduces new promising possibilities, its one-item structure causes some limitations, such as the inability to calculate Cronbach’s alpha.

## Figures and Tables

**Table 1:** Descriptive characteristics of the sample and intergroup comparisons between SIC chronotypes and effect sizes presented as eta squared (η^2^) and Cramér’s V (V).

	Morning Type (N = 30)				Evening Type (N = 64)				Highly Active type (N = 37)				Daytime Sleepy Type (N = 58)				Daytime Active Type (N = 72)				Moderately Active Type (N = 45)				ANOVA			Effect Size (η^2^ 95%CI)
	M ± SD				M ± SD				M ± SD				M ± SD				M ± SD				M ± SD				F	*p*	p*	
Age	22.13 ± 2.46				21.80 ± 2.09				21.97 ± 1.94				21.53 ± 2.30				21.60 ± 2.14				21.07 ± 1.92				1.245	0.288	0.999	0.020 (0.000; 0.045)
GHQ-30 (depression/anxiety)	22.20 ± 6.53				23.48 ± 6.71				19.59 ± 6.50 ^ce^				24.31 ± 7.06 ^b^				22.42 ± 6.43				25.27 ± 6.78 ^b^				3.662	0.003	0.045	0.058 (0.008; 0.101)
GHQ-30 (interpersonal relations)	8.73 ± 2.05				8.80 ± 2.24				8.24 ± 2.42 ^ce^				9.33 ± 2.27 ^d^				8.03 ± 1.96 ^ce^				9.71 ± 2.23 ^bd^				4.511	<0.001	0.015	0.070 (0.015; 0.117)
GHQ-30 (general functioning)	16.33 ± 3.44				16.88 ± 4.34				14.54 ± 3.83 ^ce^				17.66 ± 3.97 ^b^				16.10 ± 3.79 ^e^				18.53 ± 4.28 ^bd^				5.126	<0.001	0.015	0.079 (0.020; 0.129)
SBQ-R	5.53 ± 2.96 ^ce^				7.22 ± 3.87				7.16 ± 4.56				8.52 ± 3.96 ^ad^				6.18 ± 3.32 ^ce^				8.47 ± 3.89 ^ad^				4.626	<0.001	0.015	0.072 (0.016; 0.120)
SAQ	25.90 ± 7.92				27.70 ± 8.18				28.14 ± 9.46				31.05 ± 6.02				25.82 ± 7.46				27.84 ± 7.16				3.462	0.005	0.075	0.055 (0.006; 0.097)
																									H Kruskal–Wallis			Effect size (η^2^ 95%CI)
																									**H**	***p***	p*	
Alcohol consumption	1.10 ± 0.31				1.30 ± 0.49				1.49 ± 0.87				1.43 ± 0.68				1.51 ± 0.77				1.51 ± 0.83				7.679	0.053	0.795	0.034 (0.000; 0.078)
Sex	F	M		N	F	M		N	F	M		N	F	M		N	F	M		N	F	M		N	**Chi^2^**	***p***	p*	Effect size (V)
	73% (N = 22)	27% (N = 8)		0% (N = 0)	73% (N = 47)	27% (N = 17)		0% (N = 0)	43% (N = 16)	57% (N = 21)		0% (N = 0)	79% (N = 46)	19% (N = 11)		2% (N = 1)	60% (N = 43)	40% (N = 29)		0% (N = 0)	67% (N = 30)	33% (N = 15)		0% (N = 0)	26.118	<0.001	0.015	0.23 (0.145; 0.305)
Psychoactive substance use	Y		N		Y		N		Y		N		Y			N	Y			N	Y		N		90.052	<0.001	0.015	
	10% (N = 3)		90% (N = 27)		16% (N = 10)		84% (N = 54)		24% (N = 9)		76% (N = 28)		21% (N = 12)			79% (N = 46)	32% (N = 23)			68% (N = 49)	71% (N = 32)		29% (N = 13)					0.54 (0.472; 0.608)

M—mean; SD—standard deviation; N—number of observations; F—Fisher–Snedecor test statistics; p—probability in the test; p*—the *p*-value after Bonferroni correction; V—Cramér’s V; GHQ-30—General Health Questionnaire 30-item version; SBQ-R—Suicidal Behavior Questionnaire-Revised; SAQ—The Suicide Acceptance Questionnaire; post hoc significant intergroup differences: ^a^ vs. morning type; ^b^ vs. highly active type; ^c^ vs. daytime sleepy type; ^d^ vs. daytime active type; ^e^—vs. moderately active type; Sex coded as F = female, M = male, N = did not chose either option; Psychoactive substance use coded as Y = yes, N = no. The “yes” answer indicated that the respondent marked at least one of the exemplary substances in the following question: “What psychoactive substances do you happen to use? Cannabinoids (marijuana, hashish), Opiates (e.g., morphine, heroin), Sedative and hypnotic drugs and substances (e.g., diazepam, oxazepam, nitrazepam), Cocaine, Stimulants other than cocaine (e.g., amphetamine), Hallucinogenic substances (e.g., LSD, ecstasy, psilocybin), Androgenic-anabolic steroids”.

## Data Availability

The data that support the findings of this study are available from the corresponding author, [K.N.-D.], upon reasonable request.

## Abbreviations

GHQ-30—the 30-item General Health Questionnaire; SAQ—the Suicide Acceptance Questionnaire; SBQ-R—the Suicide Behavior Questionnaire; SIC—Single-Item Chronotyping; MEQ—the Morningness–Eveningness Questionnaire.
